# All carbon materials pn diode

**DOI:** 10.1038/s41467-018-06150-z

**Published:** 2018-09-14

**Authors:** Xiaojing Feng, Xing Zhao, Liu Yang, Mengyao Li, Fengxiang Qie, Jiahui Guo, Yuchun Zhang, Tiehu Li, Wenxia Yuan, Yong Yan

**Affiliations:** 1 CAS Key Laboratory of Nanosystem and Hierarchical Fabrication, CAS Center for Excellence in Nanoscience, National Center for Nanoscience and Technology, Beijing, 100190 China; 2 School of Chemistry and Biological Engineering, University of Science and Technology Beijing, Beijing, 100083 China; 3 School of Materials Science and Engineering, Northwestern Polytechnical University, Xi’an, 710072 China; 4 University of Chinese Academy of Sciences, Beijing, 100049 China

## Abstract

Semiconductor pn junctions are elementary building blocks of many electronic devices such as transistors, solar cells, photodetectors, and integrated circuits. Due to the absence of an energy bandgap and massless Dirac-like behaviour of charge carriers, graphene pn junction with electrical current rectification characteristics is hardly achieved. Here we show a graphene pn junction diode can be made exclusively from carbon materials by laminating two layers of positively and negatively charged graphene oxides. As the interdiffusion of oppositely charged mobile counterions, a built-in potential is created to rectify the current by changing the tunnelling probability of electrons across the junction. This graphene diode is semi-transparent, can perform simple logic operations, and since it has carbon nanotubes electrodes, we demonstrate an all carbon materials pn diode. We expect this graphene diode will expand material choices and provide functionalities (e.g. grafting recognition units on graphene oxides) beyond that of traditional semiconductor pn junctions.

## Abstract (web summary)

Chemically functionalized graphene oxide-based pn junction diodes have potential for future electronic device applications. Here, the authors report an all carbon pn diode with graphene oxide and carbon nanotubes electrodes showing excellent current rectification and efficient logic gates.

## Introduction

Graphene, an atomically-thin hexagonal lattice, has currently attracted enormous research interest because of its great promising use for a wide range including electronic devices^1,2^, energy conversion and storage^3,4^, sensors^5,6^, optics^7,8^, as well as biomedical applications^9,10^. In contrast to conventional semiconductors, its conduction and valence bands meet at Brillouin zone corners and, therefore, fabricating electronic components from this zero-gap semiconductor should find an unusual way^11^. Techniques such as cutting graphene into nanoribbons^12^, placing bias on bilayer graphenes^13^, and applying mechanical strains^14^ were developed to open a bandgap and turn off the current of graphene field-effect transistors. In addition, graphene diodes have received particular attention because diodes are the basic building blocks in a wide variety of electronic/photonic systems. Since graphene is metallic at a sufficiently large Fermi energy, Schottky diodes were fabricated by bringing metallic graphenes and semiconductor materials into contact^15,16^. With a gate modulation of localized energy levels, tunable light-emitting diode was demonstrated by using a semi-reduced graphene oxide^17^. Although pn junctions with a planar geometry could be realized by multiple electrostatic gating^18^, local chemical doping^19^, and engineering of substrates^20^, no current rectifying effect was observed owing to the Klein tunnelling of relativistic Dirac fermions^21^. In the vertical structure pn junctions, current rectifying was achieved by inserting/forming a thin layer of insulating or semiconducting barrier material between p and n graphene layers, showing behaviours of a typical resonant tunnelling diode^22,23^. So far, there is still lack of robust technique in making graphene diodes especially the diode as a similar structure to semiconductor pn junction. On the other hand, graphene oxide (GO) contains various reactive oxygen groups (–COOH, OH, and C–O–C), which provide possibility for chemical modification and produce functional materials for a variety of applications^24,25^.

Here, the graphene pn junction diode was fabricated by laminating two layers of such chemically functionalized positively (p) and negatively (n) charged graphene oxides on the transparent substrates. As the contact of two layers, mobile counterions diffused across the interface while the anchored charges on graphene oxides frame were immobile. An equilibrium condition was reached in which an internal electric field was established to tune the electron tunnelling probability through the junction. The coupled movement of ions and electrons as well as the current rectification were modelled by using a combination of Nernst–Planck diffusion and Poisson’s equations. Since carbon nanotubes were used as conductive electrodes, we demonstrated an all carbon materials pn diode. To evaluate the utility of these graphene diodes to build the processing units needed for simple computation, in combination with graphene resistors, all carbon materials AND and OR logic gates were constructed.

## Results

### All carbon materials pn diode fabrication

**Fig. 1:**
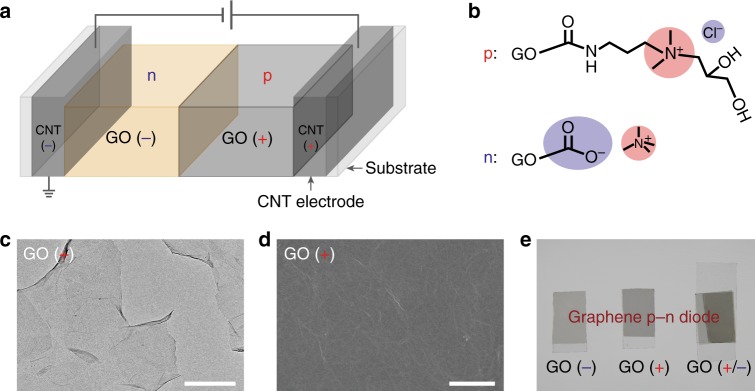
Fabrication of an all carbon materials pn diode. **a** The scheme of an all carbon materials pn diode consisting of a 40 nm-thick negatively charged GO(−) layer (n), a 40 nm-thick positively charged GO(+) layer (p), two 20 nm-thick CNT conducive electrodes, and two transparent glass supports. **b** The molecular structures of chemically functionalized positively (p) and negatively (n) charged graphene oxides. The mobile counterions are chloride anion and tetramethyl ammonium cations, respectively. **c** The typical TEM image of highly dispersed positively charged GO sheets. Scale bar, 500 nm. **d** The surface morphology (SEM image) of a 40 nm-thick positively charged GO(+) layer with a 20 nm-thick CNT layer at the bottom. Scale bar, 2 μm. **e** The photographs of negatively charged GO layer (left, 40 nm-thick), positively charged GO layer (middle, 40 nm-thick), and laminated layers (right). (Note: both positively and negatively charged GO have conductive CNT electrodes (20 nm-thick) at the bottom)

The key components of the graphene pn junction diode are a layer of negatively charged GO and a layer of positively charged GO (see Fig. 1a and Methods). Before anchoring negative and/or positive charges onto the graphene oxides, the GO was further oxidized to produce more –COOH groups which would then increase the ionic charge density in the subsequent GO layers (Supplementary Fig. 1). Negatively charged GO was synthesized by deprotonating the GO with Tetramethylammonium hydroxide solution ((CH_3_)_4_N^+^OH^−^, TMAH, pH = 10), while the positively charged GO was synthesized via a two-step procedure^26,27^ (see Methods and Supplementary Fig. 2a). The chemical functionalization was verified by the change of Zeta potential from −34.66 mV to −48.17 mV for negatively charged GO and to +39.37 mV for positively charged GO (Supplementary Table 1). Moreover, the positive modification was further demonstrated by the Fourier-transform infra-red spectroscopy (FTIR) and X-ray photoelectron spectroscopy (XPS) characterization. In FTIR, the intensities of C=O (1734 cm^−1^) and O–H (3415 cm^−1^) stretching peak were greatly suppressed while amide-I band, amide-II band, and amide-III band appeared at 1631 cm^−1^, 1460 cm^−1^, and 1384 cm^−1^, respectively, which were attributed to C=O stretching vibrations in RNH–CO and the coupling of C–N stretching vibration to N–H bending vibration (Supplementary Fig. 3). In XPS, the peaks correlated to amidation reaction (399.257 eV, C–N, 402.128 eV, C–N^+^, and 400.187 eV, N–C=O) were seen while these peaks were not observed prior to positive modification (Supplementary Fig. 4).

To fabricate the layers, carbon nanotubes (CNTs) and charged GOs solutions were successively filtered through a mixed cellulose ester membrane (MCE, pore size 0.22 μm). (Note: (1) the CNT electrodes were also chemically functionalized by the similar procedures to the GOs (see Methods) to eliminate possible dipole formation at the interface between CNT and GO layers; (2) the conductivity of charged 20 nm-thick CNT electrodes was over 10^3^ S cm^−1^, approximately 2 orders of magnitude higher than charged GOs (Supplementary Table 2); (3) both CNTs and GOs were highly dispersed in deionized water (see Fig. 1c-e, Supplementary Fig. 2, and Supplementary Fig. 5)). As the etching of MCE, the layers were transferred onto transparent glass slides and were subsequently dried by using nitrogen gas. A feature of the layers was their semi-transparent characteristics which could pass the majority of light at the wavelength 500–650 nm, specifically, approximately 70% for positively charged layer and approximately 80% for the negatively charged layer (see Fig. 1e and Supplementary Fig. 6). An all carbon materials pn diode was then fabricated by laminating the positively charged layer GO(+)/CNT(+) and the negatively charged layer GO(−)/CNT(−) with a face-to-face configuration. The graphene pn diode was finally sealed with the polydimethylsiloxane and the measurement was conducted under ambient condition with the computer-interfaced high precision electrometers.

### Electrical current rectification

**Fig. 2:**
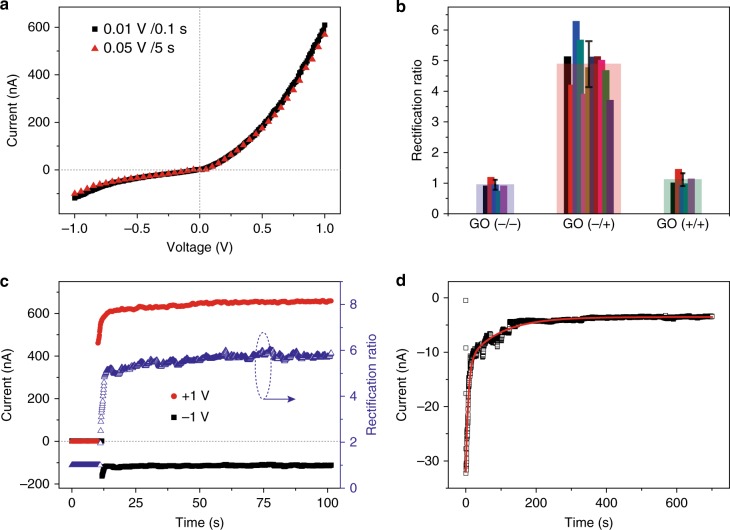
Characteization of the graphene pn diode. **a** The typical current–voltage characteristics of a graphene pn diode with fast (black square, 0.1 V/0.1 s) and slow sweep rate (red triangle, 0.05 V/5 s). **b** The statistics of rectification ratio of graphene pn diodes (*r*, approximately 5) and two control experiments composed of either GO(−/−) or GO(+/+) layers, showing no current rectification characteristics (*r*, approximately 1). The error bar for pn diode is based on 11 devices. The error bars for control experiments are based on five devices. **c** The current transients were monitored by stepping the potential from 0 to +1 V or −1 V. **d** The current transients was recorded upon laminating the positively charged and negatively charged GO layers. The current decay was fitted biexponentially

A typical current–voltage (*I*–*V*) characteristics of the graphene pn junction diode was shown in Fig. 2a. When the bias was applied on the positively charged CNT electrode (also positively charged GO side, forward bias, see Fig. 1a), the current quickly increased to a value of approximately 600 nA (at 1 V); however, a much lower current (approximately 100 nA at 1 V) was detected as the bias was applied on the negatively charged CNT electrode (reverse bias). At both fast (0.01 V/0.1 s) and slow (0.05 V/5 s) sweep rates, the current rectification ratios ($r = I_{ + 1{\mathrm{V}}}/I_{ - 1{\mathrm{V}}}$) were stabilized at approximately 6. This current rectification phenomenon was not observed in a series of control devices: (1) a device comprising of two layers of positively charged GOs, (2) a device comprising of two layers of negatively charged GOs, and (3) several other devices (see Fig. 2b and Supplementary Fig. 7 for details), which ruled out possible interface effect (e.g. Schottky barrier or dipole) between CNT electrode and GOs, and therefore, we surmised that the interface between positively and negatively charged GOs should be the key feature for the current rectification.

The current rectification effect was further verified when a bias was constantly applied (Fig. 2c). The current transients were recorded for up to 100 s by switching the bias from 0 to +1 V or −1 V. The currents were found to reach the steady state and stabilized at approximately 600 nA (+1 V) or approximately 100 nA (−1 V) shortly with application of the bias at both polarities. This demonstrated that (1) the recorded current is not dominated by the transient currents of ionic species but electrons, (2) the current rectification (blue curve, right axis of Fig. 2c), plotted in real time and stabilized at a ratio of approximately 6, is the intrinsic characteristics of the graphene pn diode where the electron tunnelling and/or hopping probability varied at forward and reverse bias.

In the GO layers, the charges anchored onto the GO frames are jammed and restricted to migration, but the counterions (Cl^−^ and (CH_3_)_4_N^+^, see Fig. 1b) are not. As the laminating of two oppositely charged GO layers, counterions diffused across the interface since it is entropically favourable. Chloride anions diffused into n region while ammonia cations diffused into p region (see p and n region in Fig. 1a), which was manifested as a transient current recorded immediately after lamination of two GO layers in the absence of an applied bias (Fig. 2d). Such interdiffusion created a potential and then transformed to an internal electric field at the junction pointing from p to n layer. This field subsequently rectified the current by facilitating or impeding the transport of electrons, depending on the polarity of external bias.

### Modelling of the pn diode

**Fig. 3:**
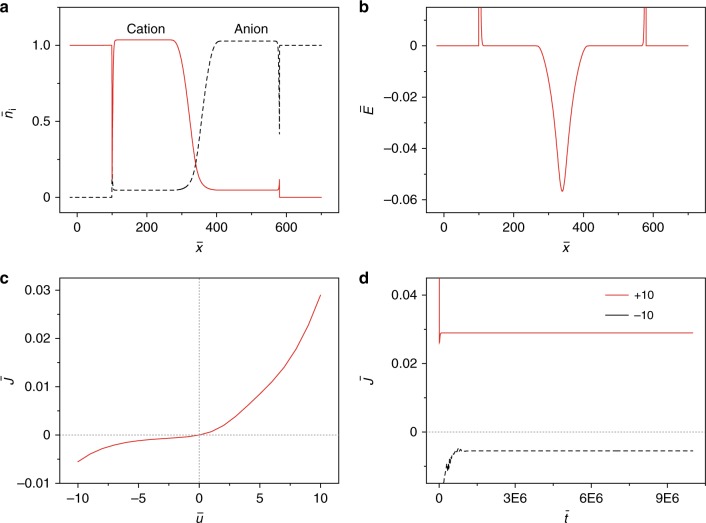
Modelling of the current rectification. **a** Spatial distribution of tetramethyl ammonium cations (red) and chloride anions (black dash) after equilibration in the absence of applied bias. **b** The internal electric field at the junction in the absence of applied bias. **c**, **d** The steady state current–voltage characteristics (**c**) and the transient current responses (**d**) at either positive or negative constant bias

The current rectification of graphene pn junction diode was explained by using a continuum charge-transport model (see details in the sections Methods and References^28,29^). This model combined Nernst–Planck diffusion and Poisson’s equations, in which both counterions and electrons could diffuse in response to concentration gradients and migrate due to local electric fields. The interdiffusion of counterions (*n*_i_, Fig. 3a), spatial evolving distribution of conduction electrons (*n*_e_, Supplementary Fig. 8c, black curve), potential (*u*, Supplementary Fig. 8d, black curve), and the internal electric field (*E*, Fig. 3b) were firstly reproduced in the absence of applied bias. By applying a forward bias, the counterions and electrons redistributed (see Supplementary Figs. 8a and 8c, red curve) and a stronger electric field (Supplementary Fig. 8e, red curve) was generated to help the electrons tunnelling from negative to positive GO layer; however, with a reverse bias, the field, on the other hand, impeded the electrons transport. Importantly, this model qualitatively reproduced (1) the current–voltage (*I–V*) characteristics (Figs. 2a and 3c), (2) the transient current responses at either positive or negative bias (Figs. 2c and 3d), and (3) the transient current as the lamination of two layers in the absence of applied bias (Fig. 2d and Supplementary Fig. 8f).

### Logic gates

**Fig. 4:**
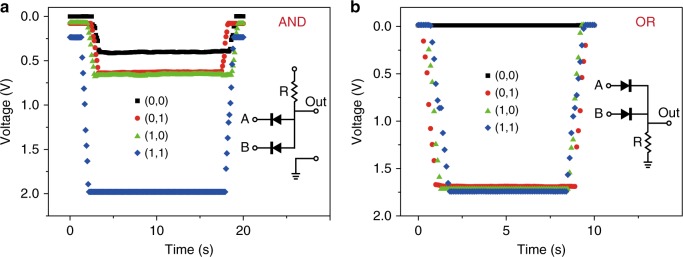
All carbon materials circuits based on graphene pn diode. **a**, **b** Demonstration of an AND logic gate (**a**) and an OR logic gate (**b**) fabricated by integrating graphene pn diodes and resistors. The inset is the circuit diagram. An input of 0 or 1 represents low (0 V) or high (2 V) potentials. For both gates, the resistance of the resistor is approximately 10 MΩ

To demonstrate the utility of the graphene pn junction diode to build the processing units needed for simple computation, AND and OR logic gates were constructed (Fig. 4). For the AND logic gate (Fig. 4a), only when a positive potential was applied to switches A and B (represented by 1), a high output potential was produced. A different circuit design allowed an OR logic gate to be realized (Fig. 4b). In contrast to the AND gate, when either of the switches A and B was activated by a high potential, the output was a high potential.

It must be noted that the cutting-off capability of the AND gate (black, red, and green curves in Fig. 4a) could not compete with the circuits based on semiconductor diodes because the graphene pn diode has a substantial internal resistance (approximately MΩ) even when a forward bias was applied (Fig. 2a, the current is in the range of sub micro-Ampere). This resistance also influenced the output of OR gate where the high potentials were less than the applied 2 V (approximately 1.7 V). Apparently, integrating a resistor with much higher resistance could improve the circuit performance. For example, with a GΩ resistor the output of the low potential in the AND gate could be less than 0.01 V and the high potential in the OR gate could be higher than 1.99 V.

## Discussion

The construction of graphene pn diode from charged GOs results in a different physical description from previous junction diodes. In a typical semiconductor pn junction, for example, the charge carriers (both holes and electrons) diffuse/drift across the interface and at the equilibrium condition, a depletion region was created and an internal electric field is established pointing from n region to p region in the absence of an external applied bias^30^. In the graphene pn diode, however, (1) the interdiffusion happened at the mixing zone rather than the depletion region in semiconductor pn diode, (2) the formation of internal electric field was entropically driven, unlike in semiconductor pn junctions where the voltage arose from equilibration of the electron chemical potential, (3) the internal electric field of graphene diode increased the probability of electron tunnelling/hopping from n to p layer, in contrast to a semiconductor pn diode where the band alignment allowed electrons to flow from n type region to p type region, but not in the reverse direction, and (4) the interdiffusion-created electric field was pointing from p to n layer while in the depletion region of a semiconductor pn diode, this field was reversed from n to p region. On the other hand, diodes based on pure ionic charge transport^31,32^ are often labelled as P or N based on the sign of conducting charge carriers: mobile cations are given as P while conducting anions are denoted by N. If positive biases are applied to the P (GO(−) with positive counterions) side of the device—i.e., forward bias—high currents are ordinarily expected. In fact, this bias corresponds to low current in the present diode. Moreover, either the device structure or rectifying principle of the graphene pn junction diode is distinct from other graphene diodes such as Schottky diodes^15,16^, light-emitting diode^17^, and resonant tunnelling diodes^22,23^. These differences strongly suggest the use of charged GOs as the electron-carrying phase leads to a different mechanism for diode behaviour than those that have been reported previously. Based on Zeta potential measurements (Supplementary Table 1) and transport characteristics (Supplementary Figs. 9–12), mimicking the definition of semiconductor pn junction, we named this new diode as graphene pn diode.

Finally, we wish to emphasize on the relatively low rectification ratio (Fig. 2b) of the graphene pn diode, and especially look for some effective methods to improve its performance. The electron tunnelling probability (either forward or reverse direction) is dependent on the magnitude of internal electric field which is directly influenced by the degree of ions mixing at the junction. Higher mobile counterions concentration would lead to more ions interdiffusion (by analogy with doping level in semiconductors) and consequently, the formation of a stronger internal electric field. Methods such as producing more –COOH groups and covalently connecting ionic dendrimers could be the most effective ways. To further increase the current rectification ratio, we propose a diode with four graphene atomic layers, which should be the lower limit of a junction diode. In such a device, two highly conductive graphene layers of electrodes are present, one is positively charged GO layer, and the last one is negatively charged GO layer. Laminating p and n layer together and removing the mobile counterions would create a very strong field by the anchored or jammed immobile charges on the GO frames, other than by the counterions in the present case. In fact, we have already demonstrated this concept by laminating two charged monolayers between gold plates instead of single sheet graphene electrodes, where the current rectification ratio improved approximately 2 orders of magnitude.

In summary, we have demonstrated a semi-transparent all carbon materials pn junction diode by laminating two layers of oppositely charged GO layers. This diode is distinct from previous devices where the current rectification happens because an internal field controls the tunnelling and/or hopping probability of conduction electrons. The observation of current rectification in graphene pn junctions greatly expands the class of materials and physical phenomena that can be used to build electronic components. Looking forward, we envision our graphene pn diode being inkjet-printed from aqueous carbon inks on flexible, transparent, and even stretchable substrates in a wide range of applications. These applications could be skin-like electronics to monitor the physiological conditions^33^, systems for recording and manipulating tissue behaviours^34^, and even smart self-powered devices with multi-functionalities^35^. Also, we wish to integrate functions of GOs (energy, sensing, catalytic, medical etc.) ^24,25^ into our graphene circuit which is hard to achieve in semiconductor electronics.

## Methods

### The synthesis of GO(−)

In a typical synthesis, 35 mL sulphuric acid (98 wt%) and 2.60 g potassium permanganate were carefully added into an ice-bath-cooled flask with 50 mL commercially available GO solution (4 mg mL^−1^, Graphenea Inc.) under constant stirring. 5 min later, the mixture solution was heated to 35 °C and kept reacting for 3 h^36^. 63 mL DI water was then poured into the mixture. 15 min later, additional 200 mL DI water and 3.5 mL hydrogen peroxide (30 wt%) were added to stop the reaction. The yellowish product was thoroughly washed with 200 mL dilute hydrochloric acid (3.5 wt%) for 5 times and then re-dispersed in DI water (150 mL, approximately 1.0 mg mL^−1^, pH = 6). This further oxidization procedure produces GOs with more –COOH groups (Supplementary Fig. 1). 1.2 mL Tetramethylammonium hydroxide (25 wt%) was finally added into the GO solution to deprotonate –COOH groups (0.25 mg mL^−1^, pH = 10).

### The synthesis of GO(+)

A two-step procedure was employed to synthesize positively charged GOs^26,27^ (Supplementary Fig. 2a). 60 mg protonated GO (the above re-oxidized), 6 mL *N,N’*-Dimethyl-1,3-propanediamine (99%), and 60 mg HATU (O–(7-Aza-1H-benzotriazol-1-yl)–*N*,*N*,*N’*,*N’*-tetramethyluronium hexafluorophosphate) were firstly mixed and reacted at 65 °C for 6 h in 60 mL *N*,*N*-Dimethylformamide (DMF). This step was repeated for three times to ensure the majority of –COOH groups were amidated. Next, 500 μL hydrochloric acid (36.5%) and 5.5 g 3-Chloro-1,2-propandiol (98 wt%) were added into an aqueous solution of above amidated GOs (50 mL, 1 mg mL^−1^). The mixture was heated to 85 °C and kept reacting for 7 h under constantly stirring and refluxing. The grayish products were washed with DI water for 4 times and finally dispersed into DI water (0.25 mg mL^−1^).

### The synthesis of CNT(−) and CNT(+)

100 mg carboxyl functionalized CNTs powder (single-walled carbon nanotubes, 2/3 semiconducting and 1/3 metallic, Chengdu Organic Chemicals Co. Ltd) was added into 100 mL mixed acid solution (3 M H_2_SO_4_ and 3 M HNO_3_ with 3 to 1 volume ratio) and refluxed for 2 h at 80 °C under stirring. This step produces CNTs with more –COOH groups. The products were collected and washed with DI water for 5 times and then re-dispersed into DI water by the assistance of Sodium deoxycholate and sonication (0.1 mg mL^−1^, pH = 6). To produce negatively charged CNTs, Tetramethylammonium hydroxide was only used (pH = 10). For the synthesis of positively charged CNTs, the same procedure with GO(+) was employed, while Hexadecyltrimethylammonium bromide (CTAB) was added to improve their dispersity.

### Graphene pn diode fabrication

The graphene pn diode was fabricated by laminating two layers of oppositely charged GO layers. To prepare GO layers, all of the carbon solutions were sonicated by a probe sonicator (150 W) for 1 h. Gradient centrifugation was then used to collect highly dispersed carbon solutions. The concentration of all carbon materials is approximately 0.03 mg mL^−1^. 15 mL CNT(−) was firstly vacuum filtered through a mixed cellulose membrane (MCE, 0.22 μm in pore size and 50 mm in diameter) and thoroughly washed with 20 mL DI water to remove surfactants. 10 mL GO(−) was subsequently added. The MCE-carbons film was peeled off and cut into desired pieces (15 mm × 10 mm) when all the carbons were dried. The MCE was then removed by floating the film onto DMF solvent for approximately 120 min and the negatively charged GO layer was transferred onto the transparent glass slide (20 mm × 14 mm). The layer was finally dried by using nitrogen gas. This produces a layer of approximately 20 nm CNTs and approximately 40 nm GOs (Supplementary Fig. 16). The same procedure was employed for preparing positively charged GO layer. Before laminating two GO layers, 50 μm diameter platinum wires were connected to the end of CNT electrodes. These are used as the leads for later electrical measurements. The two GO layers were subsequently laminated with a face-to-face configuration. This device was then wrapped by using a transparent tape and pressed by using two pieces of permanent magnet (5 mm in diameter and 1 mm in thickness) on the back sides of two substrates. To prevent atmospheric humidity from influencing the device performance, the device was encased in polydimethylsiloxane. (Note: every step should be operated carefully).

### Characterization and measurement

The structure of carbon materials was characterized by using scanning electron microscopy (SEM, Hitachi-SU8220) and transmission electron microscopy (TEM, Tecnai G2 F20 U-TWIN). The composition of GOs was evaluated by using X-ray photoelectron spectroscopy (XPS, ESCALAB250Xi) and Fourier-transform infra-red spectroscopy (FTIR, Spectrum One). Ultraviolet-visible spectroscopy (UV-vis, Lambda 950) was used to evaluate the light transmission capability. Zeta potential is tested on Zetasizer Nano ZS. The surface roughness and thickness of carbon layers were measured by using Dektak XT Stylus Profiling System. The conductivity of carbon layers was characterized by a four-probe technique (MST 4145-B). The measurement of graphene pn diode was conducted under ambient condition with a computer-interfaced high precision electrometer (Keithley 6517). To drive logic circuits, an additional 6517 electrometer was applied.

### Theory details

The graphene pn junction diode was simulated by using a continuum charge-transport model, which combined Nernst-Planck diffusion and Poisson’s equations. The details of this model was presented in references^28,29^. For simplicity, we considered one dimensional domain which contain four regions: CNT(−), GO(−), GO(+), and CNT(+). We assumed that the CNT electrode layer (either CNT(−) or CNT(+)) is equipotential^37^ and the electrons are free to move out or into the layers. The accumulation of mobile counterions in CNT layers was neglected. The flow of electrons as well as the rearrangement of ionic charges throughout the layers produced the electric current *J*. The dimensionless electric current density ($\bar J$) is given by equation (1)

1 $$\bar J = {\int} {\frac{1}{{2\bar L}}\left[ { - \left( {-\frac{{\partial \bar n_{\mathrm{e}}}}{{\partial \bar x}} + \bar n_{\mathrm{e}}\frac{{\partial \bar u}}{{\partial \bar x}}} \right) + \mathop {\sum}\limits_i {z_{\mathrm{i}}\alpha _i\beta _i} \left( { - \frac{{\partial \bar n_{\mathrm{i}}}}{{\partial \bar x}} + \bar n_{\mathrm{i}}\frac{{\partial \bar u}}{{\partial \bar x}}} \right)} \right]} {\mathrm{d}}\bar x$$

where $\bar L = L/\lambda$, *L* represents the thickness of GO(+) and GO(−) layers and $\lambda = \left[ {\frac{{\varepsilon _0\varepsilon k_{\mathrm{B}}T}}{{e^2\left( {n_{\mathrm{e}}^0 + \mathop {\sum}\limits_i {z_{\mathrm{i}}^2n_{\mathrm{i}}^0} } \right)}}} \right]^{1/2}$ is the Debye screening length; *n*_i_ and *n*_e_ are the number density of mobile ions and conduction electrons respectively, *D*_i_ and *D*_e_ are the ion and electron diffusivities, *Z*_i_ is the valence of the mobile ions, *e* is the elementary charge, *k*_B_ is the Boltzmann constant, *T* is the absolute temperature, *u* is the electrostatic potential, *ε*_0_ is the vacuum dielectric constant, and the *ε* is relative dielectric constant; $\alpha _i = \frac{{n_{\mathrm{i}}^0}}{{n_{\mathrm{e}}^0}}$, $\beta _i = \frac{{D_{\mathrm{i}}}}{{D_{\mathrm{e}}}}$, and the dimensionless variables are $\bar n_{\mathrm{i}} = \frac{{n_{\mathrm{i}}}}{{n_{\mathrm{i}}^0}}$, $\bar n_{\mathrm{e}} = \frac{{n_{\mathrm{e}}}}{{n_{\mathrm{e}}^0}}$, $\bar t = \frac{{D_{\mathrm{i}}t}}{{\lambda ^2}}$, $\bar x = \frac{x}{\lambda }$, $\bar u = \frac{{ue}}{{k_{\mathrm{B}}T}}$; $n_{\mathrm{i}}^0$ and $n_{\mathrm{e}}^0$ are the initial number density of ions and electrons respectively. The parameters we used here are $n_{\mathrm{i}}^0 = 1.8 \times 10^{22}\,{\mathrm{cm}}^{ - 3}$, $n_{\mathrm{e}}^0 = 2.6 \times 10^{20}\,{\mathrm{cm}}^{ - 3}$, *D*_i_ = *D*_e_ = 1.0 × 10^−9^ cm^2 ^s^−1^, *ε* = 4.5. The coupling Eqs.^28,29^ were then solved by using a commercial finite element solver (COMSOL 5.1).

## Electronic supplementary material

Supplementary Information

## Data Availability

The data that support the findings of this study are available from the corresponding authors upon reasonable request.

## References

[1] Novoselov KS (2004). Electric field effect in atomically thin carbon films. Science.

[2] Schwierz F (2010). Graphene transistors. Nat. Nanotechnol..

[3] Wang X, Zhi LJ, Mullen K (2008). Transparent, conductive graphene electrodes for dye-sensitized solar cells. Nano. Lett..

[4] El-Kady MF, Shao YL, Kaner RB (2016). Graphene for batteries, supercapacitors and beyond. Nat. Rev. Mater..

[5] Shao YY (2010). Graphene based electrochemical sensors and biosensors: a review. Electroanalysis.

[6] Shin SH (2017). Integrated arrays of air-dielectric graphene transistors as transparent active-matrix pressure sensors for wide pressure ranges. Nat. Commun..

[7] Liu M (2011). A graphene-based broadband optical modulator. Nature.

[8] D’Apuzzo F (2017). Terahertz and mid-infrared plasmons in three-dimensional nanoporous graphene. Nat. Commun..

[9] Yang K (2010). Graphene in mice: ultrahigh in vivo tumor uptake and efficient photothermal therapy. Nano. Lett..

[10] Yang K, Feng LZ, Hong H, Cai WB, Liu Z (2013). Preparation and functionalization of graphene nanocomposites for biomedical applications. Nat. Protoc..

[11] Castro Neto AH, Guinea F, Peres NMR, Novoselov KS, Geim AK (2009). The electronic properties of graphene. Rev. Mod. Phys..

[12] Li XL, Wang XR, Zhang L, Lee SW, Dai HJ (2008). Chemically derived, ultrasmooth graphene nanoribbon semiconductors. Science.

[13] Zhang YB (2009). Direct observation of a widely tunable bandgap in bilayer graphene. Nature.

[14] Ni ZH (2008). Uniaxial strain on graphene: Raman spectroscopy study and band-gap opening. ACS Nano.

[15] Chen CC, Aykol M, Chang CC, Levi AFJ, Cronin SB (2011). Graphene-silicon Schottky diodes. Nano. Lett..

[16] Yang H (2012). Graphene barristor, a triode device with a gate-controlled Schottky barrier. Science.

[17] Wang XM (2015). A spectrally tunable all-graphene-based flexible field-effect light-emitting device. Nat. Commun..

[18] Williams JR, DiCarlo L, Marcus CM (2007). Quantum hall effect in a gate-controlled p–n junction of graphene. Science.

[19] Lin L (2016). Tuning chemical potential difference across alternately doped graphene p–n junctions for high-efficiency photodetection. Nano. Lett..

[20] Chiu HY, Perebeinos V, Lin YM, Avouris P (2010). Controllable p–n junction formation in mono layer graphene using electrostatic substrate engineering. Nano. Lett..

[21] Young AF, Kim P (2009). Quantum interference and Klein tunnelling in graphene heterojunctions. Nat. Phys..

[22] Kim S (2013). Graphene p–n vertical tunneling diodes. ACS Nano.

[23] Britnell L (2013). Resonant tunnelling and negative differential conductance in graphene transistors. Nat. Commun..

[24] Stankovich S (2007). Synthesis of graphene-based nanosheets via chemical reduction of exfoliated graphite oxide. Carbon N. Y..

[25] Georgakilas V (2016). Noncovalent functionalization of graphene and graphene oxide for energy materials, biosensing, catalytic, and biomedical applications. Chem. Rev..

[26] Vukovic G (2009). Synthesis, characterization and cytotoxicity of surface amino-functionalized water-dispersible multi-walled carbon nanotubes. Appl. Surf. Sci..

[27] Liu WS (2015). Synthesis, characterization and antibacterial properties of dihydroxy quaternary ammonium salts with long chain alkyl bromides. Chem. Biol. Drug. Des..

[28] Yan Y, Warren SC, Fuller P, Grzybowski BA (2016). Chemoelectronic circuits based on metal nanoparticles. Nat. Nanotechnol..

[29] Nakanishi H (2011). Dynamic internal gradients control and direct electric currents within nanostructured materials. Nat. Nanotechnol..

[30] Sze, S. M. & Ng, K. K. *Physics of Semiconductor Devices* (John Wiley & Sons, 2007).

[31] Bernards DA, Flores-Torres S, Abruña HD, Malliaras GG (2006). Observation of electroluminescence and photovoltaic response in ionic junctions. Science.

[32] Cayre OJ, Chang ST, Velev OD (2007). Polyelectrolyte diode: nonlinear current response of a junction between aqueous ionic gels. J. Am. Chem. Soc..

[33] Wang SH (2018). Skin electronics from scalable fabrication of an intrinsically stretchable transistor array. Nature.

[34] Dai X, Hong G, Gao T, Lieber CM (2018). Mesh nanoelectronics: seamless integration of electronics with tissues. Acc. Chem. Res..

[35] Li W (2017). Nanogenerator-based dual-functional and self-powered thin patch loudspeaker or microphone for flexible electronics. Nat. Commun..

[36] Jankovsky O (2016). A new member of the graphene family: graphene acid. Chem. Eur. J..

[37] Orphanou A, Yamada T, Yang CY (2012). Modeling of a carbon nanotube ultracapacitor. Nanotechnology.

