# Supplementary material for: All carbon materials pn diode
Source: Nat Commun. 2018 Sep 14;9:3750. doi: 10.1038/s41467-018-06150-z (PMC6138713; doi:10.1038/s41467-018-06150-z)
Supplement: Supplementary file 1 — Supplementary Information [file 41467_2018_6150_MOESM1_ESM.pdf]

Supplementary Information

All carbon materials pn diode

Xiaojing Feng[1,2†], Xing Zhao[1,3†], Liu Yang[1†], Mengyao Li[1,4], Fengxiang Qie[1], Jiahui Guo[1,4], Yuchun Zhang[1], Tiehu Li[3], WenxiaYuan[2], and Yong Yan[1*]

[1]*CAS Key Laboratory of Nanosystem and Hierarchical Fabrication, CAS Center for Excellence in Nanoscience, National Center for Nanoscience and Technology, Beijing 100190, China*

[2]*School of Chemistry and Biological Engineering, University of Science and Technology Beijing, Beijing, 100083, China*

[3]*School of Materials Science and Engineering, Northwestern Polytechnical University, Xi'an 710072, China*

[4]*University of Chinese Academy of Sciences, Beijing, 100049, China*

[†]*These authors contributed equally to this work*

*Correspondence to: yany@nanoctr.cn

Supplementary Figures

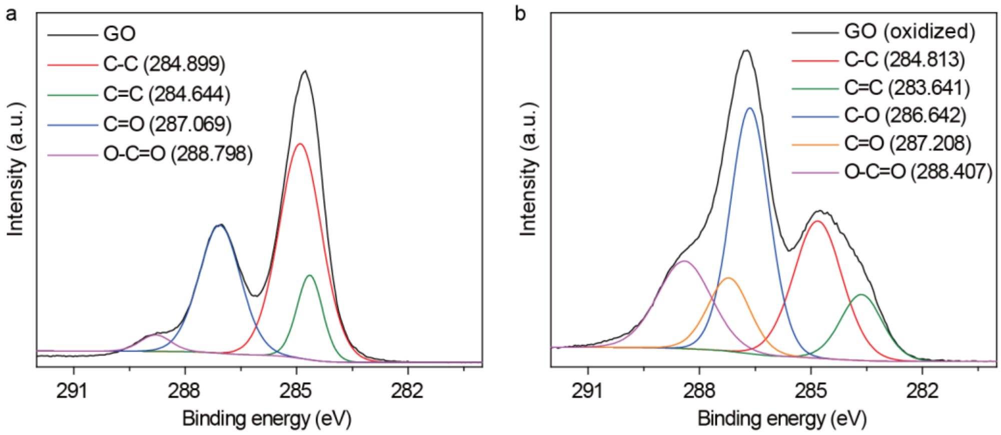

Supplementary Figure 1. X-ray photoelectron spectroscopy (XPS) of (a) commercial GOs and (b) further oxidized GOs. In commercial GO, peaks 284.899 (C-C, 53.39%), 284.644 (C=C, 12.66%), 287.069 (C=O, 31.02%), 288.798 (O-C=O, 2.94%) were observed; In further oxdized GO, peaks 284.813 (C-C, 25.04%), 283.641 (C=C, 10.27%), 286.642 (C-O, 35.70%), 287.208 (C=O, 11.39%), 288.407 (O-C=O, 17.60%) were observed. The ratio of -COOH group has dramatically increased from 2.94% (left, magenta curve) to 17.60% (right, magenta curve).

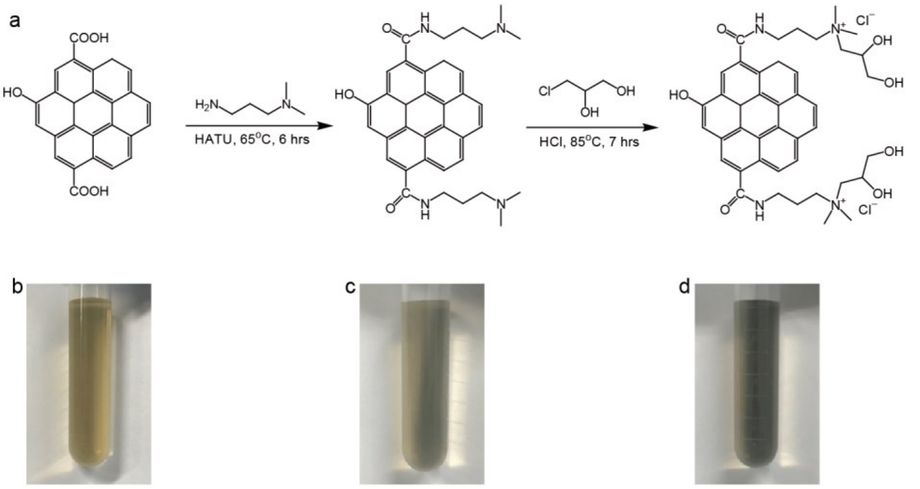

Supplementary Figure 2. The synthesis of positively charged GO. (**a**) Chemical reactions for the synthesis of GO (+). (**b**) to (**d**) The photos of commercial available

GO, amidated GO, and charged GO aqueous solution. The color of GO solutions gradually changed from yellowish to grayish.

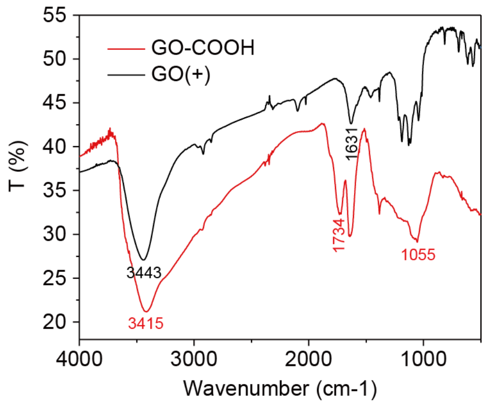

Supplementary Figure 3. Fourier-transform infrared spectroscopy (FTIR) of GOs (red curve) and positively charged GOs (black curve). For GO, the peak at 3415 cm^{-1} was attributed to the O-H stretching bands of hydroxy and carboxylic acid moieties. The peak at 1734 cm^{-1} and was attributed to C=O stretching bands of carboxylic acids and the peak at 1055 cm^{-1} was attributed to the C-O bonds. For GO (+), the intensity of O-H peak was suppressed and a new peak of 3443 cm^{-1} which contributed to O-H and N-H stretching vibrations appeared. A new peak at around 2920 cm^{-1} could be attributed to stretching vibrations of methyl, methylene and methines. The peak of C=O stretching vibrations in RNH-CO shifted to 1631 cm^{-1} compared to C=O in COOH groups, which overlapped the signal of C=C bands on aromatic rings, namely, amide-I band; also, amide-II band and amide-III band appeared at 1460 cm^{-1} and 1384 cm^{-1} which were attributed to coupling of C-N stretching vibrations and N-H bending vibrations. In the fingerprint region, C-O stretching peaks in –CH(OH)- (1114 cm^{-1}) and $–CH_2(OH)$ (1044 cm^{-1}) appeared in the spectrum and two new peaks at 614 cm^{-1} and 570 cm^{-1} could be attributed to C-N

(O=C-N) bending vibrations.

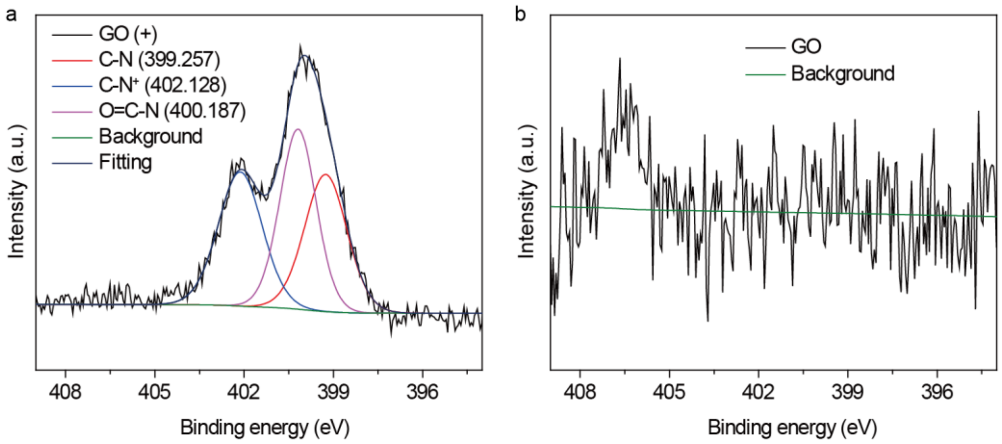

Supplementary Figure 4. X-ray photoelectron spectroscopy of (a) positively charged GOs and (b) GOs prior to positive functionalization. In the positively charged GO, peaks from amidation reaction, 399.257 (C-N), 402.128 ($C-N^+$), and 400.187 (N-C=O) were appeared, while in the GO, these peaks were not observed.

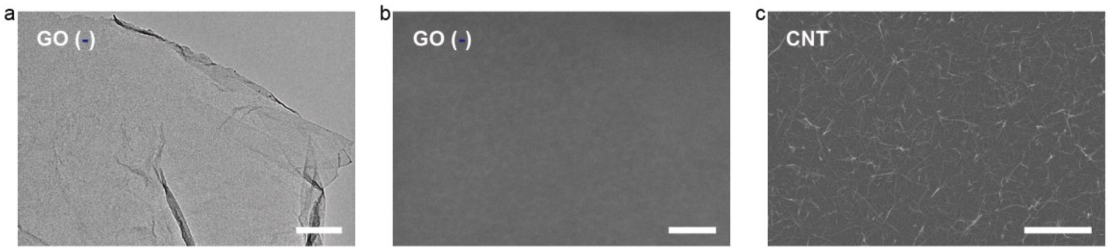

Supplementary Figure 5. Structural characterization of GOs and CNT electrode. (**a**) and (**b**) Transmission electron microscopy (TEM) and scanning electron microscopy (SEM) images of negatively charged GO and GO layer. Scale bars, 100 nm for a and 200 nm for b. (**c**) SEM image of CNT electrode. Scale bar, 2 μm.

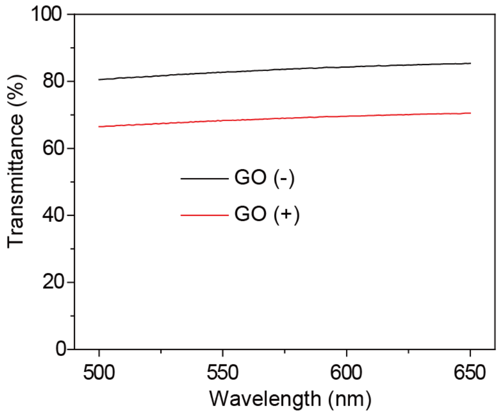

Supplementary Figure 6. Ultraviolet-visible spectroscopy (UV-vis) of positively (red) and negatively (black) charged GO layer. The transmittance characteristics of GO layers was examined by using UV-vis at wavelength from 500 to 650 nm. The majority of light was passed through GO layers, specifically, 68% and 83% for GO (+) and GO (-) layers respectively at a wavelength of 550 nm.

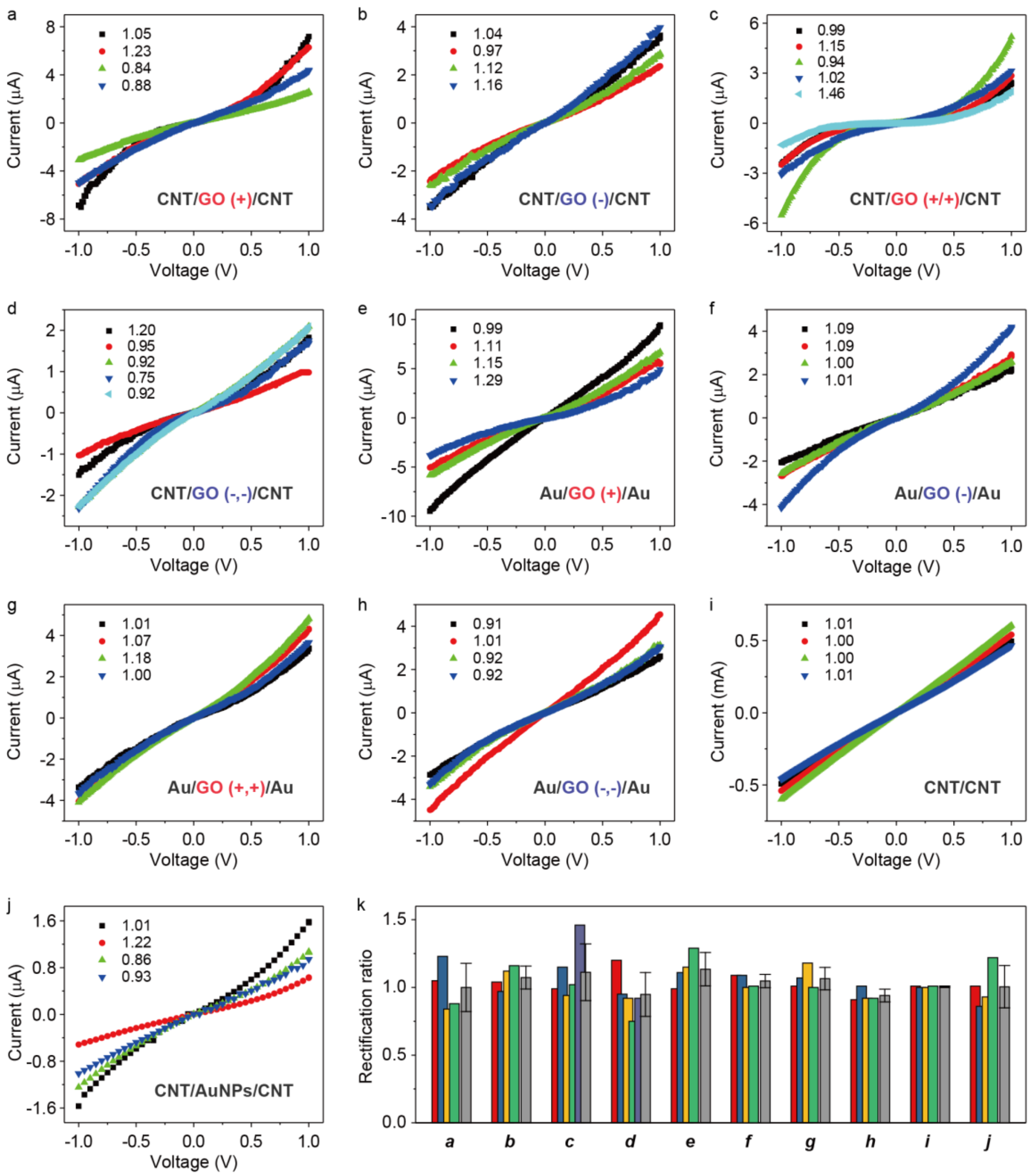

Supplementary Figure 7. Current-voltage characteristics of ten control experiments. (**a**) Laminating one layer of positively charged GO, (**b**) one layer of negatively charged GO, (**c**) two layers of positively charged GO, and (**d**) two layers of negatively charged GO between two CNT electrodes. (**e**) to (**h**) Four control devices were fabricated by using gold electrodes. (**i**) The device was fabricated by laminating two CNT electrodes into direct contact, and (**j**) a layer of alkylthiol-stabilized gold nanoparticles was inserted into two CNT electrodes. In contrast to the graphene pn

diode (Fig. 2a), the current ratio (rectification ration, $r = I_{+1V} / I_{-1V}$, supplementary equation 1) is *ca.* 1 (**k**), no clear current rectification effect has been found. Most error bars are based on 4 devices except for c and d in which the error bars are based on 5 devices.

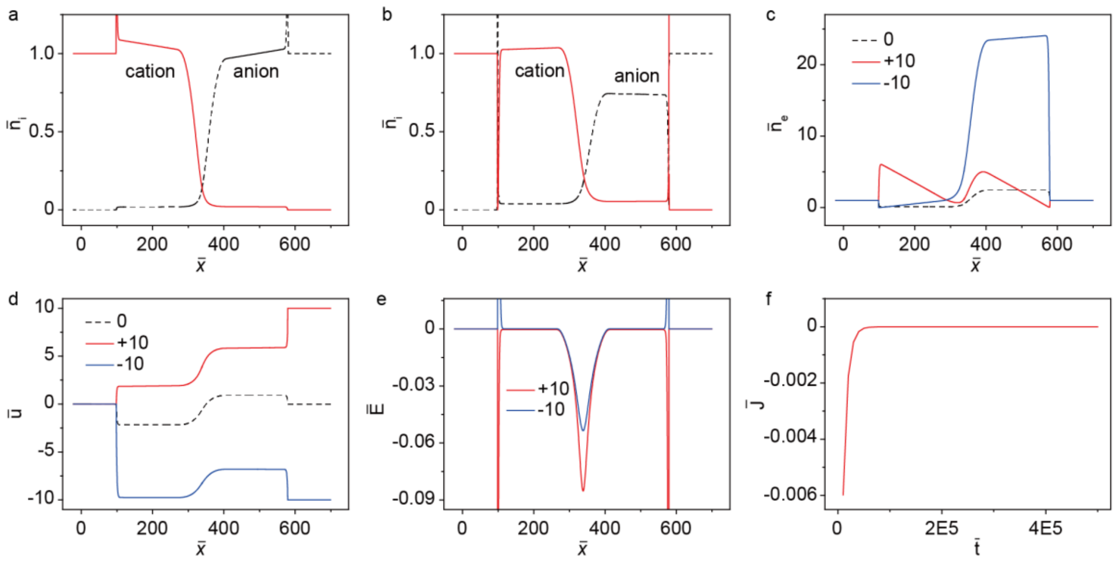

Supplementary Figure 8. Modelling of the graphene pn diode. Spatial distribution of tetramethyl ammonium cations (red) and chloride anions (black dash) by applying (**a**) forward and (**b**) reverse bias. (**c**) Distribution of electrons, (**b**) potentials, and (**e**) electric fields in the absence of applied bias (black dashed curves), forward biases (red curves), and reverse biases (blue curves). (**f**) The transient current recorded as the lamination of two GO layers.

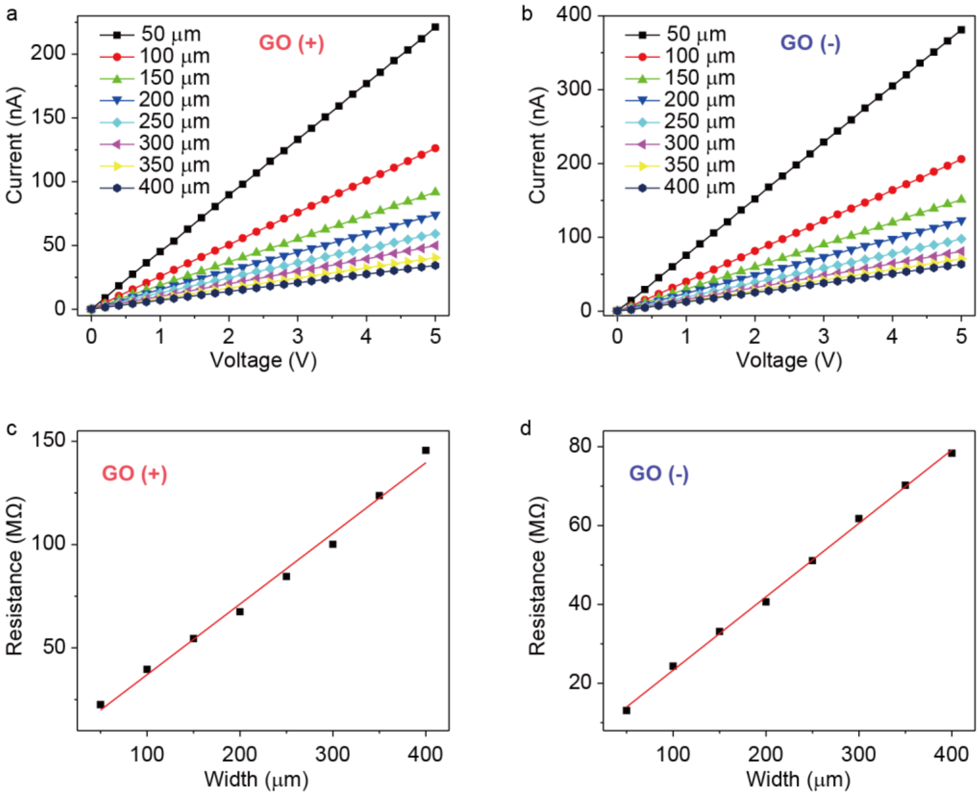

Supplementary Figure 9. Channel length dependent electrical characteristics of GO layers. (**a**) and (**b**), The current-voltage characteristics of GO (+) and GO (-) layers at different channel lengths, respectively. (**c**) and (**d**), The resistance of GO (+) and GO (-) layers at different channel lengths, respectively.

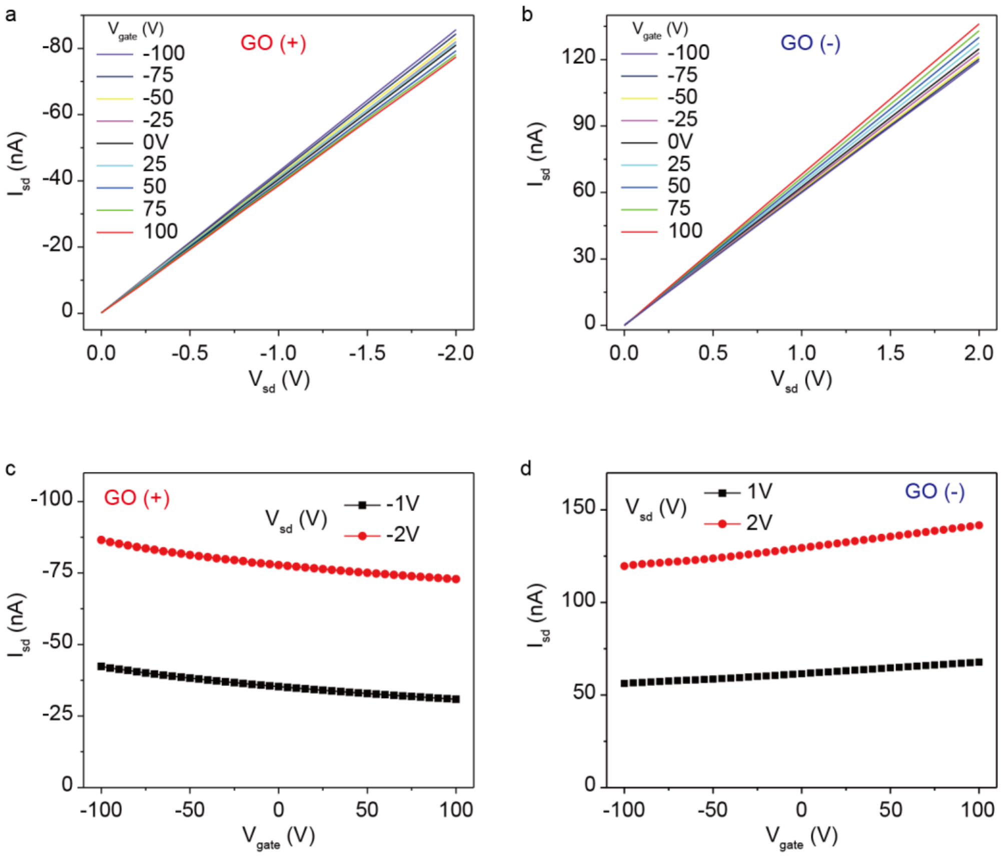

Supplementary Figure 10. Transport properties of GO (+) and GO (-) layers. (a) and (**b**), The output characteristics of GO (+) and GO (-) field-effect transistors (FET) with a gate voltages varied from -100 V to 100 V, respectively. (**c**) and (**d**) The transfer characteristics of GO (+) and GO (-) FETs at the bias of 1 V and 2 V (V_{ds}). The channel length is 50 μm and the thickness of SiO_2 layer is 300 nm.

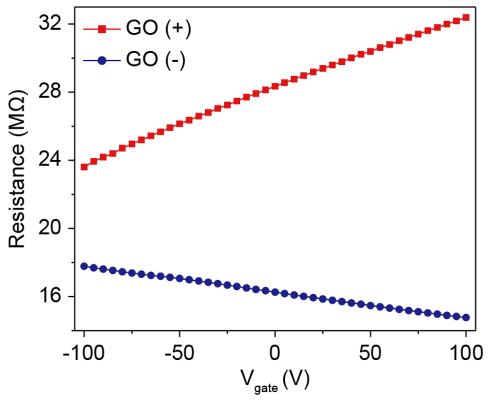

Supplementary Figure 11. The correlation between the GO layer resistance and

the gate voltage. Applying the positive gate voltage, the conductivity of GO (+) layer has decreased while the conductivity of GO (-) layer has increased. These behaviour indicated that the GO (+) is p type while the GO (-) is n type materials.

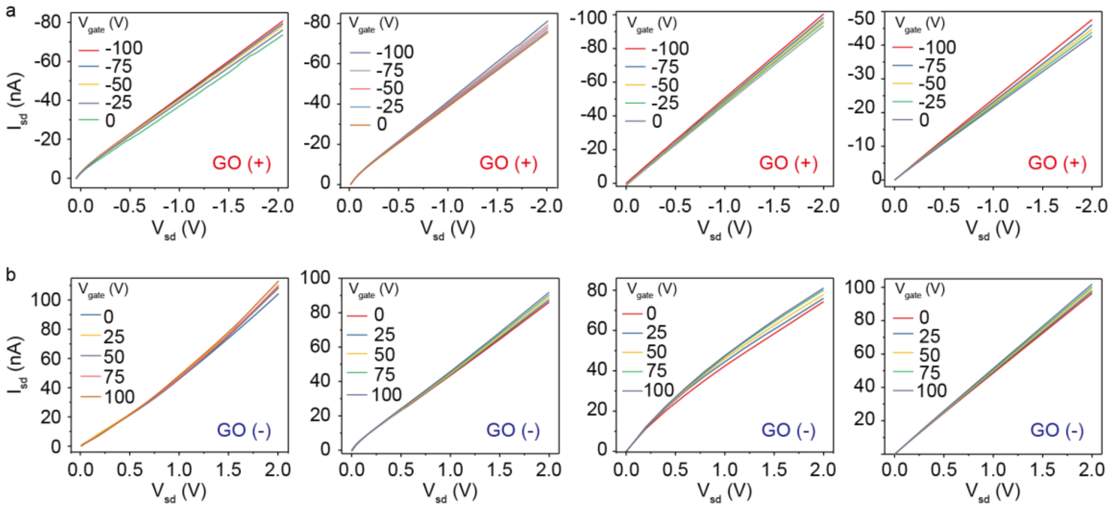

Supplementary Figure 12. The output performance of additional eight FET devices fabricated by using GO (+) and GO (-) layers. The channel length is 50 μm.

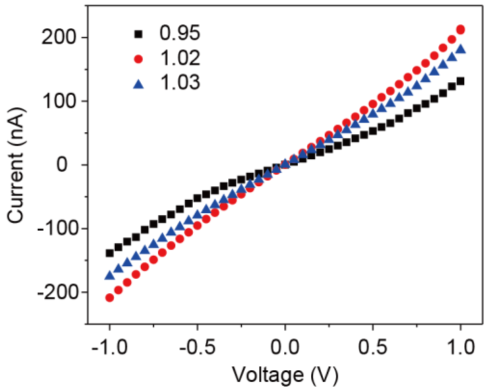

Supplementary Figure 13. Current-voltage characteristics of a device fabricated by laminating GO (+) and GO (-) layers at 2% humidity. No rectifying effect was observed because the interdiffusion of mobile counterions was greatly suspended and no apparent internal electric field was established at the interface. Note, under 2%

humidity, the output performance of GO-based FETs has no change.

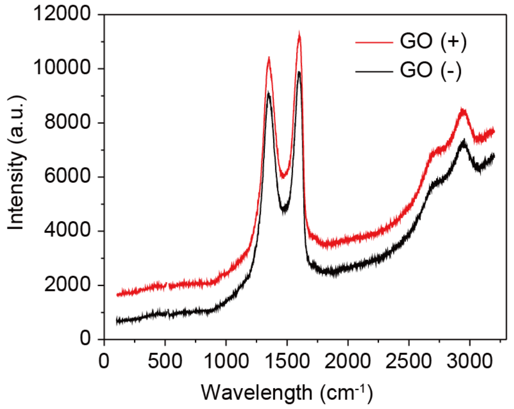

Supplementary Figure 14. The Raman spectrum of GO (+) and GO (-) layers. Both GO layers displayed a strong D band where the intensity ratios between D and G band (I_D/I_G) were approximately 0.96 due to presence of large number of chemical and structural defects. However, no significant difference in terms of the number of defects was observed for positively and negatively charged GO layers. Moreover, the same batch of GO (Graphenea Inc.) was used to synthesize positively and negatively charged GOs. These indicated that the concentration of counterion ions in positively charged GO layer is commensurate with that in negatively charged GO layer.

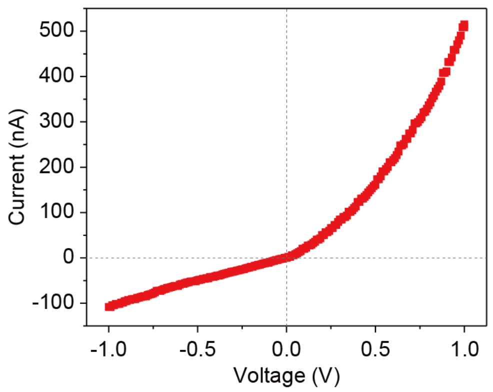

Supplementary Figure 15. Current-voltage characteristics of a device fabricated by laminating GO (+) and GO (-) layers on sputtering gold electrodes. A similar

rectifying effect was observed in this device. A rectification ratio of approximately 5 was calculated which is close to the value of CNT-based device. This control experiment further ruled out significant effect of CNT on the performance of graphene pn diode.

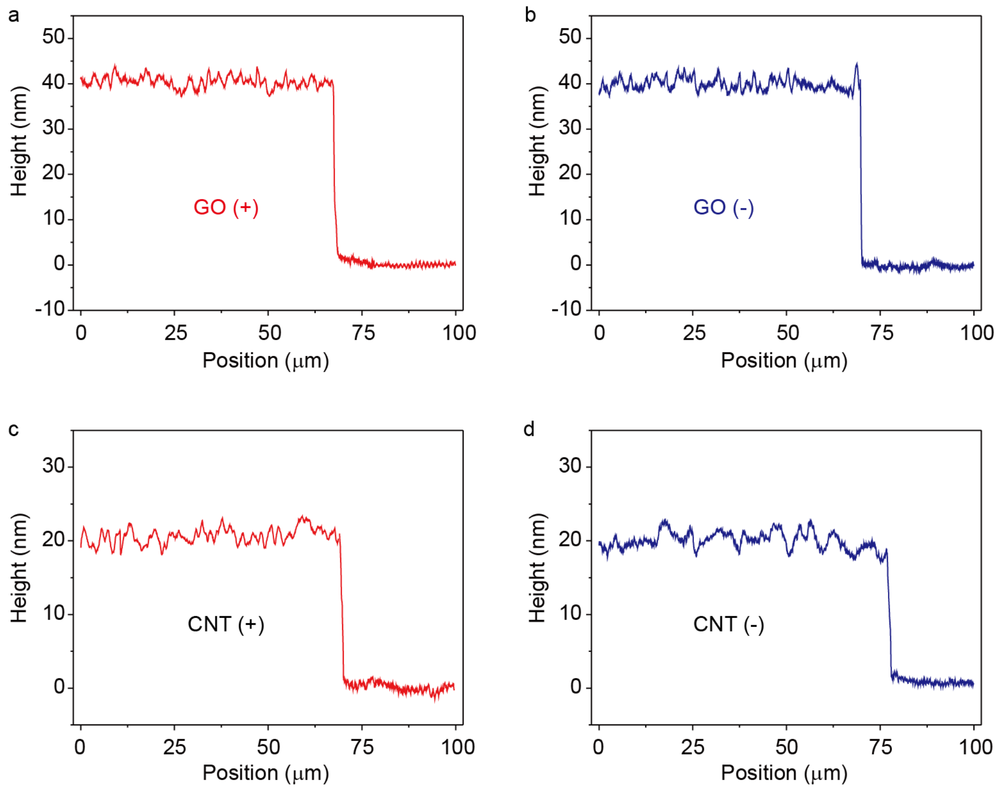

Supplementary Figure 16. The thickness and surface roughness GOs and CNT electrodes characterized by Dektak XT Stylus Profiling System (Bruker). (**a**) and (**b**) Height profile of GO layers. (**c**) and (**d**) Height profile of CNT electrodes.

Supplementary Tables

	GO (-)	GO (+)	CNT (-)	CNT (+)
Zeta potential (mV)	-48.17	39.37	-31.57	60.87

Supplementary Table 1. The Zeta potentials of all carbon materials.

	GO (-)	GO (+)	CNT (-)	CNT (+)
Thickness (nm)	40	40	20	20
Sheet Resistance (Ω/□)	45K	46K	340	420
Conductivity (S/cm)	5.5	5.38	1470	1190

Supplementary Table 2. The conductivity and thickness of carbon layers.
